# A highly heterogeneous HIV-1 epidemic in the Central African Republic.

**DOI:** 10.3201/eid0203.960310

**Published:** 1996

**Authors:** M Massanga, J Ndoyo, D J Hu, C P Pau, S Lee-Thomas, R Hawkins, D Senekian, M A Rayfield, J R George, A Zengais, N N Yatere, V Yossangang, A Samori, G Schochetman, T J Dondero

**Affiliations:** Ministère de la Santé Publique et de la Population, Central African Republic.


### A Highly Heterogeneous HIV-1 Epidemic in the Central African Republic

The Central African Republic has been strongly affected by the human immunodeficiency virus (HIV) epidemic. Although itself sparsely populated, the republic borders five other African countries and is crossed by the trans-African highway, which connects West and Central Africa with East Africa (Figure 1). Wide genetic variation reported among HIV-1 subtypes (on the basis of small numbers of samples) suggests multiple introductions of HIV into the republic (1). However, except in small studies and unpublished reports (2), the distribution and serologic reactivities of HIV within the Central African Republic have not been characterized.

The need to conduct HIV surveillance has also been highlighted by reports of highly divergent strains of HIV-1 group O from neighboring Cameroon (3,4) and by recent findings that some of these strains were not reliably detected by current antibody screening tests (5-7). To assess the prevalence and distribution of HIV in the Central African Republic, the local Ministry of Health AIDS Program, assisted by the Centers for Disease Control and Prevention (CDC), initiated a nationwide sentinel surveillance survey in late 1994. Sentinel surveillance has been useful for obtaining information on HIV infection (8). In particular, serial sentinel studies have permitted the spread of HIV to be monitored and trends to be followed over time. In this report, we present findings on the distribution of HIV-1 infections and serologic reactivities of HIV in the Central African Republic.

From October through December 1994, a nationwide sentinel surveillance survey was conducted in 10 cities and towns: Bambari, Bangassou, Bangui, Berberati, Bossangoa, Bozoum, Bria, Gamboula, M'Baiki, and Mobaye (Figure 1). The two primary population groups included were women attending prenatal clinics and male and female attendees at sexually transmitted disease (STD) clinics. Two additional sites in the capital city, Bangui, were included to perform testing for laborers and university students.

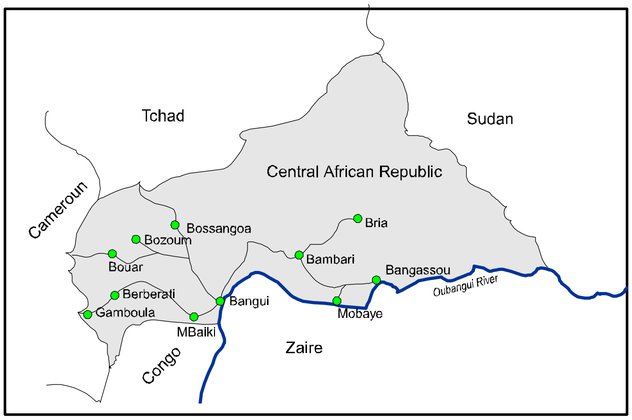

Figure 1. Map of the Central African Republic with location of sentinel sites.

Serum samples, anonymous and without any personal identifiers, were collected and transported to the National Public Health Laboratory in Bangui. Testing for this study was supported by CDC, which used U.S. Public Health Service guidelines for confirmation (9). All sera were tested with the Genetic Systems HIV-1/2-enzyme immunoassay (EIA) kit and all repeat positive sera were confirmed by HIV-1 Western blot (Cambridge Biotech). Specimens positive by initial EIA but negative for HIV-1 by Western blot or indeterminate were further tested for HIV-2 antibodies by an HIV-2 Western blot (Cambridge Biotech). In addition, samples from 11 sites (with sufficient quantity) that were positive by initial EIA were serotyped by using peptides representing the known HIV-1 subtypes A-F and the divergent HIV-1 group O (10,11).

A total of 2,259 persons were tested from 17 sites from 10 cities and towns. Between 2.7% and 30.7%, by site, were positive for HIV-1 by repeat EIA and Western blot confirmation (Table). A higher HIV-1 prevalence (25.3% to 30.7%) was observed among STD clinic attendees, whereas the prevalence among women at prenatal care clinics was generally >5% and as high as 16.7% (the exception was the lower rate in women from the prenatal care clinic in Gamboula. No HIV-2 infection was detected among the 175 persons whose serum was initially positive by EIA but negative for HIV-1 by Western blot or indeterminate by Western blot for HIV-2.

Among 247 samples serotyped by peptide EIA, 173 were HIV-1 positive by Western blot, 60 were indeterminate, and 14 were HIV-1 negative (Figure 2). No divergent HIV-1 group O infection was found among these persons. Serotype C (24%) was most prevalent among the monoreactive specimens. Many of the specimens were reactive to multiple peptides; 16 (9%) did not recognize any of the peptides and may represent more divergent HIV-1 strains.

The high seroprevalences (Table) indicate that HIV-1 infection is a serious public health problem in a number of groups in the Central African Republic. The wide variation of HIV-1 prevalence even within similar groups in a region illustrates the heterogeneous nature of this epidemic. The HIV-1 prevalence was especially high among STD clinic attendees. Although the number of specimens from STD clinic attendess was relatively small, the high proportion of HIV-infected persons at all four STD clinics is a cause for concern. Because Bangui and Bambari are major crossroads for transport in the Central African Republic as well as on the east-west highway to other countries, the potential for further HIV spread is great.

Since STDs are associated with (and may facilitate) HIV transmission (12), STD diagnosis and control are integral components of HIV prevention. CDC, with financial support from the U.S. Agency for International Development, recently helped establish two pilot STD treatment sites in Bambari and Bria.

Although HIV-2, which is endemic to West Africa, has not been reported from the Central African Republic, reports of HIV-1 Group O from neighboring Cameroon highlighted the importance of monitoring these strains. Although no HIV-2 or divergent HIV-1 group O infections were found in this study, the extremely wide pattern of serologic reactivity among HIV-1-infected persons suggests a very heterogenous distribution of HIV-1 strains in the republic. This is confirmed by the wide variety of genotypes (subtypes) found in the republic in the past (1), and suggests that in certain populations, at least, multiple subtypes of HIV-1 have been introduced over time (7). This finding may be important to the development of effective future HIV vaccines for use in this region.

Since the quantity of specimens collected in this large-scale survey was not sufficient for genetic analysis, further studies are needed to characterize the genetic diversity of HIV in the Central African Republic. Given the limited search for HIV variants and the diversity of HIV subtypes recognized thus far, both a wider distribution of the HIV strains and the existence of yet more divergent variants seem likely (7). For these reasons, CDC plans to assist the Republic's National AIDS Control Program with further characterization of virus for divergent subtypes; these findings indicate the need for more resources to help the program maintain and expand HIV and STD prevention.

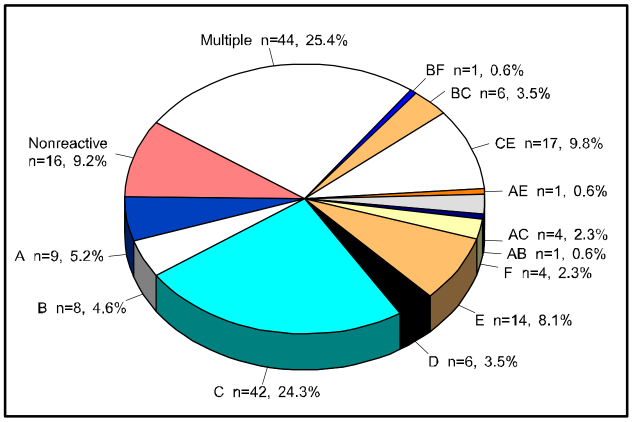

Figure 2. Serotype distribution among 173 HIV-1-infected persons. Letters indicate serologic reactivities to peptides to the given HIV-1 subtypes A-F. Sera reactive to two different subtype peptides are given as a combination of two letters. Multiple refers to sera that reacted to more than 2 different subtype peptides. Nonreactive refers to sera that did not react to any of the peptides used.

Table. Prevalence of HIV-1 by sentinel site/population

| Sentinel site/Population | Positive by Western blot (%) | Total |
|---|---|---|
| Bambari | | |
| Prenatal clinic women | 20 (13.3) | 150 |
| STD clinic attendees | 23 (26.7) | 86 |
| Bangassou | | |
| Prenatal clinic women | 16 (10.0) | 160 |
| Bangui | | |
| Prenatal clinic women A | 20 (13.3) | 150 |
| Prenatal clinic women B | 8 ( 5.3) | 150 |
| STD clinic attendees | 20 (25.3) | 79 |
| University students | 14 ( 9.3) | 150 |
| Workers | 23 (15.4) | 149 |
| Berberati | | |
| Prenatal clinic women | 16 (10.7) | 150 |
| Bossangoa | | |
| Prenatal clinic women | 9 ( 6.0) | 148 |
| Bozoum | | |
| Prenatal clinic women | 20 (13.2) | 151 |
| STD attendees | 23 (30.7) | 75 |
| Bria | | |
| Prenatal clinic women | 22 (16.7) | 132 |
| STD attendees | 24 (30.4) | 79 |
| Gamboula | | |
| Prenatal clinic women | 4 ( 2.7) | 150 |
| M'Baiki | | |
| Prenatal clinic women | 8 ( 5.3) | 150 |
| Mobaye | | |
| Prenatal clinic women | 9 ( 6.0) | 150 |
| Total number tested | | 2,259 |

### Acknowledgments

We thank Jacob Ngaba, Harold Jaffe, Gérard Grezenguet, Abdoulaye Kozemaka, David Gittelman, Lucienne Gaba, Françoise Jabot, David Espey, Benoit Soro, Robert Gribbin, Samuel Laeuchli, and the staff of the American Embassy, Bangui, Central African Republic.

**Marcel Massanga,* Justin Ndoyo,* Dale J. Hu,† Chou-Pong Pau,† Stephanie Lee-Thomas,† Reginald Hawkins,† Dominique Senekian,* Mark A. Rayfield,† J. Richard George,† Amédée Zengais,* Noel Ngalla Yatere,* Victor Yossangang,* Aliou Samori,* Gerald Schochetman,† and Timothy J. Dondero†**

*Ministère de la Santé Publique et de la Population, Central African Republic; †Centers for Disease Control and Prevention, Atlanta, Georgia, USA

### References

1. Murphy E, Korber B, Georges-Courbot MC, You B, Pinter A, Cook D, et al. Diversity of V3 region sequences of human immunodeficiency viruses type 1 from the Central African Republic. AIDS Res Hum Retroviruses 1993;9:997-1006.
2. Mathiot CC, Lepage C, Chouaib E, Georges-Courbot MC, Georges AJ. HIV seroprevalence and male to female ratio in central Africa [letter]. Lancet 1990;335:672.
3. DeLeys R, Vanderborght B, Vanden Haesevelde M, Heyndrickx L, van Geel A, Wauters C, et al Isolation and partial characterization of an unusual human immunodeficiency retrovirus from two persons of West-Central African origin. J Virol 1990;64:1207-16.
4. Gurtler LG, Hauser PH, Eberle J, von Brunn A, Knapp S, Zekeng L, et al. A new subtype of human immunodeficiency virus type 1 (MVP-5180) from Cameroon. J Virol 1994;68:1581-5.
5. Loussert-Ajaka I, Ly TD, Chaix ML, Ingrand D, Saragosti S, Courouсé AM, et al. HIV-1/HIV-2 seronegativity in HIV-1 subtype O infected patients. Lancet 1994;343:1393-4.
6. Schable C, Zekeng L, Pau CP, Hu D, Kaptue L, Gurtler L, et al. Sensitivity of United States HIV antibody tests for detection of HIV-1 group O infections. Lancet 1994; 344:1333-4.
7. Hu DJ, Dondero TJ, Rayfield MA, George JR, Schochetman G, Jaffe HW, et al. The emerging genetic diversity of HIV: the importance of global surveillance for diagnostics, research, and prevention. JAMA 1996; 275:210-6.
8. Slutkin G, Chin J, Tarantola D, Tarantola D, Mann J. Sentinel surveillance for HIV infection: a method to monitor HIV infection trends in population groups. WHO/GPA/DIR/88.8. Geneva: WHO, 1988.
9. Centers for Disease Control. Public Health Service guidelines for counseling and antibody testing to prevent HIV infections and AIDS. MMWR 1987;36:509-15.
10. Pau CP, Kai M, Holloman-Candal DL, Luo CC, Kalish ML, Schochetman G, et al. Antigenic variation and serotyping of HIV type 1 from four World Health Organization-sponsored HIV vaccine sites. AIDS Res Human Retroviruses 1994;10:1369-77.
11. Pau CP, Hu DJ, Spruill C, Schable C, Lackritz E, Kai M, et al. Surveillance for HIV-1 Group O infections in the United States. Transfusion 1996;36:398-400.
12. Laga M, Nzila N, Goeman J. The interrelationship of sexually-transmitted diseases and HIV infection: implications for the control of both epidemics in Africa. AIDS 1991;5(suppl 1):S55-S63.
